# White-Nose Syndrome Fungus: A Generalist Pathogen of Hibernating Bats

**DOI:** 10.1371/journal.pone.0097224

**Published:** 2014-05-12

**Authors:** Jan Zukal, Hana Bandouchova, Tomas Bartonicka, Hana Berkova, Virgil Brack, Jiri Brichta, Matej Dolinay, Kamil S. Jaron, Veronika Kovacova, Miroslav Kovarik, Natália Martínková, Karel Ondracek, Zdenek Rehak, Gregory G. Turner, Jiri Pikula

**Affiliations:** 1 Institute of Vertebrate Biology, Academy of Sciences of the Czech Republic, Brno, Czech Republic; 2 Department of Botany and Zoology, Masaryk University, Brno, Czech Republic; 3 Department of Ecology and Diseases of Game, Fish and Bees, University of Veterinary and Pharmaceutical Sciences, Brno, Czech Republic; 4 Environmental Solutions & Innovations Inc., Cincinnati, Ohio, United States of America; 5 Institute of Biostatistics and Analysis, Masaryk University, Brno, Czech Republic; 6 Administration of the Moravian Karst Protected Landscape Area, Nature Conservation Agency of the Czech Republic, Blansko, Czech Republic; 7 Pennsylvania Game Commission, Harrisburg, Pennsylvania, United States of America; Southern Illinois University, United States of America

## Abstract

Host traits and phylogeny can determine infection risk by driving pathogen transmission and its ability to infect new hosts. Predicting such risks is critical when designing disease mitigation strategies, and especially as regards wildlife, where intensive management is often advocated or prevented by economic and/or practical reasons. We investigated *Pseudogymnoascus [Geomyces] destructans* infection, the cause of white-nose syndrome (WNS), in relation to chiropteran ecology, behaviour and phylogenetics. While this fungus has caused devastating declines in North American bat populations, there have been no apparent population changes attributable to the disease in Europe. We screened 276 bats of 15 species from hibernacula in the Czech Republic over 2012 and 2013, and provided histopathological evidence for 11 European species positive for WNS. With the exception of *Myotis myotis*, the other ten species are all new reports for WNS in Europe. Of these, *M. emarginatus, Eptesicus nilssonii, Rhinolophus hipposideros, Barbastella barbastellus* and *Plecotus auritus* are new to the list of *P. destructans*-infected bat species. While the infected species are all statistically phylogenetically related, WNS affects bats from two suborders. These are ecologically diverse and adopt a wide range of hibernating strategies. Occurrence of WNS in distantly related bat species with diverse ecology suggests that the pathogen may be a generalist and that all bats hibernating within the distribution range of *P. destructans* may be at risk of infection.

## Introduction

Host-pathogen dynamics represent a balance between the pathogen's ability to infect and the host's ability to resist, with an intensive arms race between the two reflected in co-evolutionary adaptations. With host switching, pathogens temporarily escape the arms race. New, naive hosts may show lower resistance and other characteristics favourable to the pathogen. Overlapping distribution of a pathogen and its potential host(s) is key to host switching driven by opportunity [1]. The spread of emerging wildlife pathogens may have economic consequences, even in species indirectly linked to humans [2]. Fungal infections in amphibians and bats that result in population declines [3], for example, can lead to increased agricultural costs where humans chemically compensate for ecosystem services provided by these organisms in terms of insect control.

White-nose syndrome (WNS) is an emerging disease of hibernating bats associated with skin infection by *Pseudogymnoascus [Geomyces] destructans*, a recently recognised fungal pathogen [4]–[7]. Severe skin damage results in disruption of torpor pattern, premature depletion of fat reserves and mortality in affected bats in North America [8]. High mortality rates at affected localities and the rapid spread of the infection since 2006 continues to threaten bat diversity [9]–[11].

While WNS has characteristics of an epizootic, gradually expanding through North American hibernacula from its original detection site [12], [13], *P. destructans* is pan-European in distribution [14], [15]. Aside from seasonality in the appearance of white fungal growth [15], detailed spatio-temporal data for *P. destructans* infection in Europe are lacking. Fortunately, mass mortality has not been observed in European bats to date [14].

WNS can be transmitted either directly through bat-to-bat contact or indirectly through contact with pathogen propagules in the environment [6], [16] and the infection's spread is assumed to be both density- and frequency-dependent [10]. Multiple factors, such as hibernation in large assemblages or length of hibernation season, play a role in the epidemiology of this fungal disease and ecological and behavioural characteristics of bat species may affect the risk of infection [3]. Traits such as selection of hibernaculum roost sites with differing microclimatic conditions and solitary versus gregarious hibernation behaviour may also influence the impact of WNS [10]. Other risk factors associated with hibernating bat mortality in North America include distance from the first WNS-affected site, cluster size, species diversity and composition and type of hibernaculum [13].

Prior to 2012, bat species positive for *P. destructans* in North America included *Myotis austroriparius, M. grisescens, M. leibii, M. lucifugus, M. septentrionalis, M. sodalis, M. velifer, Perimyotis subflavus* and *Eptesicus fuscus* [11], [16], [17]. Dermatohistopathology has revealed fungal infection with cupping erosions and skin invasion diagnostic for WNS in a *M. myotis* specimen hibernating in the Moravian Karst, Czech Republic [18] and eight species (*M. myotis, M. oxygnathus/M. blythii, M. brandtii, M. daubentonii, M. dasycneme, M. mystacinus, M. nattereri* and* M. bechsteinii*) have been reported positive for the WNS fungus in Europe based on direct microscopy of characteristic *P. destructans* conidia, fungal culture and genetic analysis [14], [15], [19]–[22]. Photographic evidence of fungal growth suggests that *M. emarginatus, E. nilssonii* and *Rhinolophus hipposideros *may also prove positive for *P. destructans* [14].

In summary, a total of 17 vespertilionid bat species had been reported positive for the WNS fungus in North America and Europe prior to 2012 and, as the epizootic spreads through North America and surveillance continues in Europe, it is expected that the number of infected species will increase. Hereinafter, the term *P. destructans*-infected or -positive relates to those species for which the fungal pathogen has been confirmed by laboratory methods such as fungal culture and genetic analysis. WNS-positive represents those species where the infection has been diagnosed through characteristic histopathological lesions, such as fungal hyphae densely packed in so-called cupping erosions and/or invasion of the dermis [23].

Little is known about *P. destructans* infection in European bat species less abundant or less commonly observed in hibernacula. Knowledge of pathological effects associated with the WNS fungus in European bat species is even poorer. While it is a commonly held view that European bats are more resistant or resilient than those in North America [17], our monitoring revealed three further species positive for *P. destructans* infection in 2012. Differences in their hibernation behaviour and taxonomy inspired us to examine the ecological and behavioural traits and phylogeny of European and North American species reported positive for *P. destructans* in order to identify any similarities in behaviour and habitat use and to identify any other species that may be at risk.

First, we examined the hypothesis that more bat species are positive for WNS in Europe than currently reported via histopathology, considered as the ‘gold standard’ for diagnosing WNS [23], [24]. Second, we hypothesised that ecology, behaviour and phylogenetic relationships of hibernating bat species influence risk of infection by *P. destructans*. Aside from ecological similarities, those species most often found positive for *P. destructans* and WNS belong to the genus *Myotis*, indicating that phylogenetic relatedness of hosts may facilitate invasion by the fungus. To test this, we compared the ecological and behavioural traits of hibernating bats from Europe and North America. We grouped species with similar behaviour and habitat use and used confirmed positive species to propose possible additional species susceptible to infection. We constructed a phylogeny of vespertilionids and rhinolophids from Europe and North America, to test the hypothesis that infected bats are phylogenetically closely related. Finally, we screened species of unknown infection status in Czech hibernacula to test the validity of our models and predictions.

Here, we provide histopathological evidence of multiple European species positive for WNS. We found that infected bat species are ecologically diverse, utilising a range of hibernating and feeding strategies. Although bat species previously described as being *P. destructans*-positive have been phylogenetically related, the pattern begins to break down with the newly diagnosed taxa; the data presented herein demonstrating that the host range for this fungal pathogen is more diverse than previously realized.

## Materials and Methods

### Ethics statement

The Czech Academy of Sciences' Ethics Committee has reviewed and approved Animal Use Protocol No. 169/2011 in compliance with Law No. 312/2008 on Protection of Animals against Cruelty, as adopted by the Parliament of the Czech Republic. Bats were monitored for WNS and presence of the causative agent *P. destructans* in the spring of 2012 and 2013 in caves of the Moravian Karst, mines near Mala Moravka in the Jeseniky Mountains, and in the Podyji National Park, all in the Czech Republic. Non-lethal sampling was in compliance with Law No. 114/1992 on Nature and Landscape Protection and was based on permits 01662/MK/2012S/00775/MK/2012, 866/JS/2012 and 00356/KK/2008/AOPK issued by the Nature Conservation Agency of the Czech Republic. Bats were handled so as to minimise stress and duration of sampling procedures between capture and release. Numbered aluminium rings were attached around the forearm for long-term identification prior to release at the site.

### Screening bat species in Czech hibernacula for *P. destructans* infection

When screening bats for WNS and *P. destructans* we 1. captured bats emerging from hibernacula at the end of the hibernation season using mist nets, 2. swabbed the wing membrane for fungal culture using the Fungi-Quick transport system (Copan Innovation, Italy), 3. briefly illuminated the bats with a flashlight to detect any visible fungal growth, 4. trans-illuminated the wing membrane using ultraviolet light (UV; wavelength 368 nm) to detect any WNS lesions, 5. photographed wing membranes of each bat under both visible and UV light, 6. took a wing punch biopsy from all WNS-suspected skin lesions (i.e. areas of orange-yellow fluorescence) using a sterile and disposable 4 mm skin biopsy punch (Kruuse, Denmark), 7. used polymerase chain reaction (PCR) to confirm *P. destructans* from fungal cultures or skin swabs using the FLOQSwabs system (CopanFlock Technologies, Italy), and 8. undertook complete histopathological examinations of skin samples.

A total of 276 bats were screened for WNS and *P. destructans* and 123 skin biopsies were taken for histological examination from 15 bat species (Table 1).

**Table 1:** Bats examined for white-nose syndrome and *Pseudogymnoascus destructans* infection in Czech hibernacula (Europe).

Species	Screened	Biopsied	Histo+	WNS prevalence (%)	St. error
*Myotis myotis* a	67	56	37	55.22	6.08
*Myotis daubentonii^b^*	25	13	4	16.00	5.76
*Myotis bechsteinii^b^*	21	7	2	9.52	6.78
*Myotis nattereri^b^*	20	8	3	15.00	7.10
*Myotis brandtii^b^*	17	1	1	5.88	8.23
*Myotis alcathoe^b^*	8	7	0	0	15.94
*Myotis emarginatus^b^*	39	7	5	12.82	5.35
*Rhinolophus hipposideros^b^*	28	5	1	3.57	5.18
*Eptesicus nilssonii^b^*	4	1	1	25.00	26.89
*Plecotus auritus^c^*	23	11	5	21.73	8.60
*Barbastella barbastellus^c^*	17	3	3	17.64	8.24
*Plecotus austriacus*	3	1	0	0	32.22
*Eptesicus serotinus*	2	1	0	0	39.61
*Pipistrellus pipistrellus*	1	1	0	0	48.47
*Myotis dasycneme^b^*	1	1	1	100	48.47
**Total**	**276**	**123**	**63**	**22.82**	**2.53**

a =  species reported positive for WNS fungus prior to 2012, *^b^*  =  species recognised as positive in 2012, *^c^*  =  bat species recognised as positive in 2013.

Screened  =  numbers of bats captured and examined using UV light trans-illumination to detect WNS lesions, biopsied  =  numbers of bats biopsied due to WNS-suspected skin lesions viewed under UV light, histo+  =  specimens positive for WNS diagnostic features under histopathological examination (i.e. cupping erosions and fungal invasion of dermis), WNS prevalence  =  percentage of bats positive on histopathology out of the total number screened.

Formalin-fixed punch biopsy samples were embedded in paraffin and serial 5 µm tissue sections were prepared and stained for fungi with periodic acid–Schiff stain. Histopathological findings were classified as WNS based on previously described diagnostic criteria [23]. Samples collected to cultivate fungi were transferred onto Petri dishes containing Sabouraud agar, sealed with parafilm, inverted and incubated in the dark at 10 °C. Pure fungal cultures were established from fungal growth developing at 14 days or later. *Pseudogymnoascus destructans* was confirmed through characteristic asymmetrically curved conidia via direct microscopy [5]. Fungal isolates or skin swabs were further identified using PCR and follow-up sequencing of amplicons [25] and real-time PCR [24]. A pure culture of *P. destructans* isolate grown at 10 °C on Sabouraud agar and genetically confirmed (EMBL-Bank accession number: HE588133; [18]) served as a PCR control.

### Analysis of European and North American bat ecological traits

The list of European and North American bat species was prepared according to Simmons [26]; those species included in the study being those with complete or partial distribution in continental Europe or North America for which data are available. In total, we reviewed ecological and behavioural variables for 87 species. Of these, 47 were assessed for all variables and were subjected to ecological modelling analysis.

Eleven traits were chosen to describe bat species: 1. Infection status (*P. destructans*-positive/*P. destructans*-negative), 2. Cave or non-cave hibernation, 3. Region (Palearctic/Nearctic distribution), 4. Clustering during hibernation (clustering/non-clustering; i.e. hibernating in groups where multiple individuals touch), 5. Temperature preference (thermophilic/cryophilic; mainly according to Webb *et al.* [27]), 6. Preferred roost type during hibernation (exposed/hidden/both), 7. Size of clusters during hibernation (no clustering/small clusters < 50 bats/medium clusters of 51 to 500 bats/large clusters > 500 bats), 8. Distribution range (very large area/large area of approximately half a continent/moderate size/small area/very small area; according to Horáček *et al.* [28]), 9. Food (dominant insect food group represented by Diptera/Lepidoptera/Coleoptera/generalists), 10. Foraging habitat (open/edge/closed), 11. Body size (small - up to 5 g/medium - 5 to10 g/large - over 10 g).

These traits, which were assessed based on a primary literature review and expert evaluation, were chosen as those most likely to influence susceptibility of bats to WNS, the spread of *P. destructans* infection or survival rate during hibernation [10]. As the disease can inflict long-term damage to affected bats surviving the hibernation season, other medically relevant factors may also influence survival and reproduction in the active season [29]. Categorical variables were coded as *n* – 1 binary, dummy variables, where *n* is the number of categories. Data for the traits of each bat species are provided in Table S1. Grouping of ecologically similar species was performed via neighbour-joining clustering of squared Euclidean distances using ape in R language [30], [31].

### Phylogenetic reconstruction of European and North American bats

The phylogeny of bats from Europe and North America was extracted from a maximum likelihood phylogenetic tree using Phylocom version 4.2 [32]. The complete phylogeny of the Vespertilionidae, Miniopteridae and Cistugidae families (from which the tree used here was pruned) was reconstructed from a concatenated DNA sequence matrix of 13 mitochondrial and nuclear genes with 64% of missing data (Table S2). Three *Rhinolophus* species were used as an outgroup. Phylogeny was reconstructed using the partitioning scheme suggested by the greedy algorithm, utilising the Bayesian Information Criterion assessment in PartitionFinder [33]. The tree space was searched using maximum likelihood analysis with automatic majority-rule bootstopping option [34]. By extracting the target species' phylogeny from a comprehensive tree, we were able to obtain a phylogeny that exploits currently available diversity to optimise relationships and branch lengths, and thus mitigate possible analysis artefacts.

### Statistical analysis and hypothesis testing of *P. destructans* infection occurrence

We explored the distribution pattern of *P. destructans* infection on a tree based on bat trait variation and molecular phylogeny. Occurrence of *P. destructans* infection represents a presence/absence variable, rather than a continuous trait, meaning that it is suitable for community structure analysis. The phylogenetic signal for explanatory variables and for *P. destructans* infection was calculated in Phylocom using the comstruct function. In order to assess relatedness of species that share a specific characteristic, mean phylogenetic distance (MPD) and mean nearest phylogenetic taxon distance (MNTD) were compared to the null model, which assumes random dispersal of the trait on the tree. MPD measures the mean branch length between two randomly selected taxa from a sample, and is calculated as the sum of branch lengths to the node representing their most recent common ancestor. MNTD is the mean branch length between a taxon within the sample and its nearest relative. The null model randomised samples across phylogeny in 9,999 replicates. The distribution of the trait on a tree is clustered if values of MPD and MNTD obtained are higher than 95% of values obtained from the null samples standardised by the standard deviation of the null samples. The comparison is expressed as net relatedness index (NRI) and nearest taxon index (NTI) greater than zero [32]. Clustered distribution of a trait or phylogenetic signal means that species that share the trait are more closely related to one another than to a random taxon sampled from the tree.

Species' ecological and behavioural characteristics often show a heritable component such that close relatives have similar traits [35]. Such characteristics might then be adaptive and their evolution further decoupled from the assumption of sample independence needed for general statistical approaches. The evolutionary relationships of traits in our dataset were removed from comparisons by using phylogenetic generalised least squares (PGLS) in the Caper package of R [36]. We used a variance-covariance matrix calculated from the phylogeny with branch lengths transformed according to the Ornstein-Uhlenbeck model in geiger [37]. The PGLS model was developed via a step-down procedure, using the Akaike Information Criterion (AIC) to compare alternative models.

## Results

### Screening species with unknown infection status in Czech hibernacula

**Figure 1:**
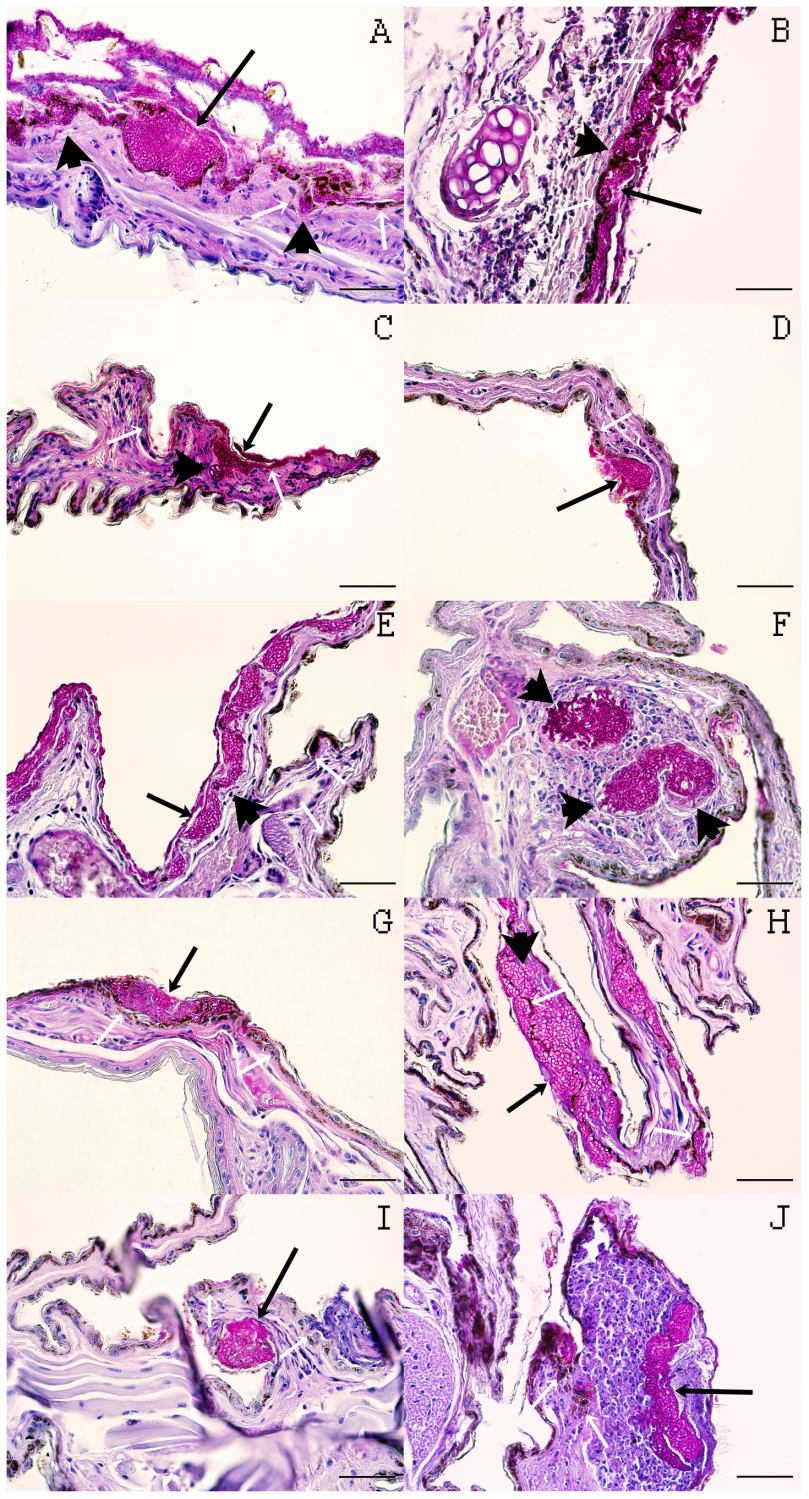
Histopathological skin lesions consistent with white-nose syndrome in ten European bat species. (A) *Myotis emarginatus*, (B) *Eptesicus nilssonii*, (C) *Rhinolophus hipposideros*, (D) *Plecotus auritus*, (E) *Barbastella barbastellus*, (F) *M. dasycneme*, (G) *M. nattereri*, (H) *M. daubentonii*, (I) *M. bechsteinii*, (J) *M. brandtii*. The photographs illustrate i) extensive infection of the wing membrane and cup-shaped epidermal erosions (A, E, H, J; long black arrow); ii) cup-like epidermal erosions in the pinna (B; long black arrow), iii) *Pseudogymnoascus destructans* hyphae obscuring the basement membrane and invading the dermis (A, B, C, E, H; black arrow); iv) a single cupping erosion packed with fungal hyphae in the wing membrane (C, D, G, I; long black arrow); v) colonisation of a hair follicle by *P. destructans*, fungal hyphae present in the associated sebaceous gland and regional connective tissue (F; black arrow); vi) marked signs of inflammation (B, F, J); and vii) a cellular inflammatory crust that sequesters fungal hyphae (A, J). White arrows within each photograph indicate the interface between epidermis and dermis. Periodic acid-Schiff stain; scale bar  =  50 µm. *M. myotis* not shown because WNS lesions in this species have already been documented elsewhere [18].

We tested a broad diversity of European hibernating bats for *P. destructans* infection and skin lesions pathognomonic for WNS. Analysis of 123 skin biopsy samples collected in 2012 and 2013 revealed histopathological findings matching criteria used for diagnosis of WNS in 63 bats (22.82% prevalence; Table 1) of 11 species, i.e. *M. myotis, M. daubentonii, M. bechsteinii, M. nattereri, M. brandtii, M. emarginatus, M. dasycneme, E. nilssonii, R. hipposideros, B. barbastellus* and *P. auritus* (Figure 1). With the exception of *M. myotis*, the other ten species are all new reports of WNS in Europe. Of these, *M. emarginatus, E. nilssonii, R. hipposideros, B. barbastellus* and *P. auritus* are new to the list of *P. destructans*-infected bat species. Fungus isolates or skin swabs from histopathologically positive bats were identified as *P. destructans* using PCR.

### Risk of *P. destructans* infection in bat species of unknown infection status

#### Testing the hypothesis of phylogenetic relatedness

**Figure 2:**
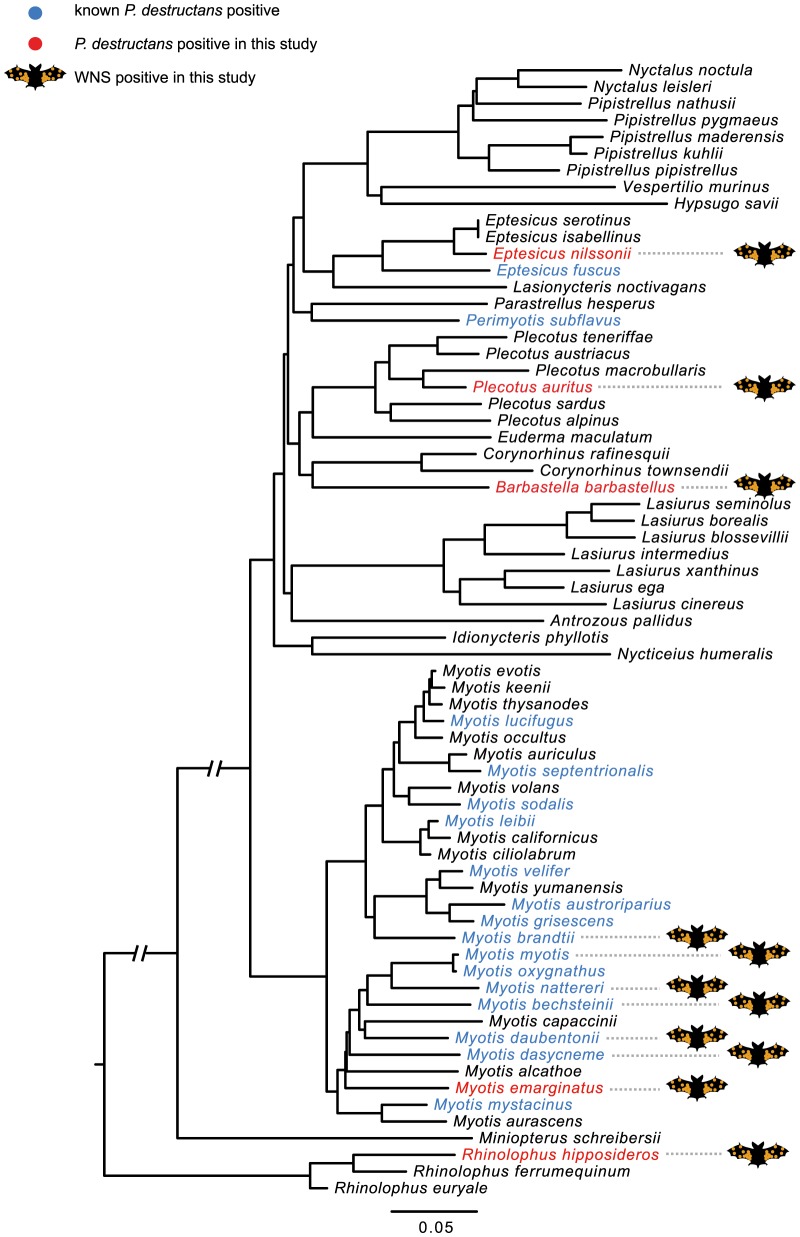
Phylogenetic reconstruction of bats from Europe and North America. The reconstruction was based on a concatenated DNA sequence matrix from 13 loci, purged from a maximum likelihood vespertilionid phylogeny rooted on *Rhinolophus*. Blue  =  species reported positive for WNS fungus prior to 2012, red  =  species recognised positive in this study, bat image  =  bat species diagnosed as WNS positive in this study.

*P. destructans*-infected species were clustered together by molecular phylogeny (MPD  =  0.212, NRI  =  2.913, *p* < 0.001), meaning that pairs of infected species were, on average, more closely related than random species pairs from Europe and North America. When sister species or nearest relatives were considered, however, our results indicated that infection of both, either or neither was random (MNTD  =  0.111, NTI  =  1.556, *p*  =  0.06; Table 2, Figure 2). Nine explanatory variables showed NRI and/or NTI not equal to zero, indicating that phylogenetic comparative methods were needed due to relatedness of taxa with shared traits (Table 2).

**Table 2:** *Pseudogymnoascus destructans* infection in relation to chiropteran phylogeny and ecological similarity.

Variable	*N*	MPD	NRI	*p*_NRI_	MNTD	NTI	*p*_NTI_
*Explanatory*							
CAVE	42	0.270	1.300	0.105	**0.091**	**1.984**	**0.025**
REGION	23	0.332	−1.606	0.944	0.114	1.125	0.135
CLUSTER	27	**0.241**	**2.151**	**0.012**	0.109	1.180	0.124
TEMPERATURE	21	0.318	−1.020	0.841	0.128	0.569	0.292
SHELTERhidden	31	**0.246**	**2.147**	**0.013**	0.121	0.115	0.450
SHELTERexposed	21	0.316	−0.929	0.814	0.119	1.004	0.165
SHELTERboth^a^	5	0.224	1.051	0.106	0.170	0.754	0.229
CSIZEno	20	0.337	−1.639	0.947	0.125	0.736	0.240
CSIZEsmall	7	**0.126**	**3.025**	**0.001**	**0.082**	**2.474**	**0.004**
CSIZEmedium	9	0.280	0.201	0.474	0.162	0.404	0.358
CSIZElarge	11	0.265	0.592	0.306	0.166	0.064	0.488
RANGEverylarge	15	0.333	−1.217	0.875	0.188	−1.328	0.902
RANGElarge	13	0.311	−0.565	0.708	**0.213**	**−1.872**	**0.970**
RANGEmoderate	14	0.246	1.189	0.112	0.133	0.859	0.203
RANGEsmall	5	**0.098**	**2.865**	**0.001**	**0.059**	**2.675**	**0.001**
FOODcolleoptera	5	0.258	0.482	0.330	0.169	0.706	0.249
FOODdiptera	9	0.237	1.093	0.117	0.144	0.902	0.188
FOODgeneralist	18	0.313	−0.763	0.770	0.128	0.772	0.232
FOODlepidoptera	14	0.286	0.117	0.475	0.147	0.355	0.368
FOODother	1	n/a					
HABITATclosed	11	0.280	0.228	0.458	0.142	0.802	0.220
HABITATopen	13	0.314	−0.623	0.724	0.178	−0.652	0.739
HABITATedge	23	0.267	0.855	0.211	**0.093**	**2.321**	**0.007**
BODYsmall	10	0.292	−0.097	0.576	0.142	0.855	0.204
BODYmedium	21	**0.211**	**2.829**	**0.001**	**0.105**	**1.698**	**0.048**
BODYlarge	16	**0.368**	**−2.259**	**0.989**	0.149	0.070	0.475
*Response*							
*Pd*+ on phylogeny (Figure 2)	20	**0.212**	**2.914**	**0.001**	0.111	1.555	0.058
*Pd*+ on 'eco' tree (Figure 3)	20	7.839	0.862	0.201	4.356	0.277	0.384

a =  species using both types of shelters are also included in the previous categories.

Phylogenetic signal of explanatory variables on a phylogeny of bats from Europe and North America and of *P. destructans* infection on both phylogeny and a neighbour-joining tree based on squared Euclidean distances of ecological and behavioural traits of hibernating bat species. Values in bold indicate significant clustering or over-dispersion of *P. destructans* infection on the tree. Note that all categories of explanatory variables were tested here, but *n* - 1 dummy variables were included in the PGLS model. *N*  =  number of species scored positive for the given variable, MPD  =  mean phylogenetic distance, NRI  =  net relatedness index, MNTD  =  mean nearest taxon phylogenetic distance, NTI  =  nearest taxon index.

#### Testing the hypothesis of ecological similarity

**Figure 3:**
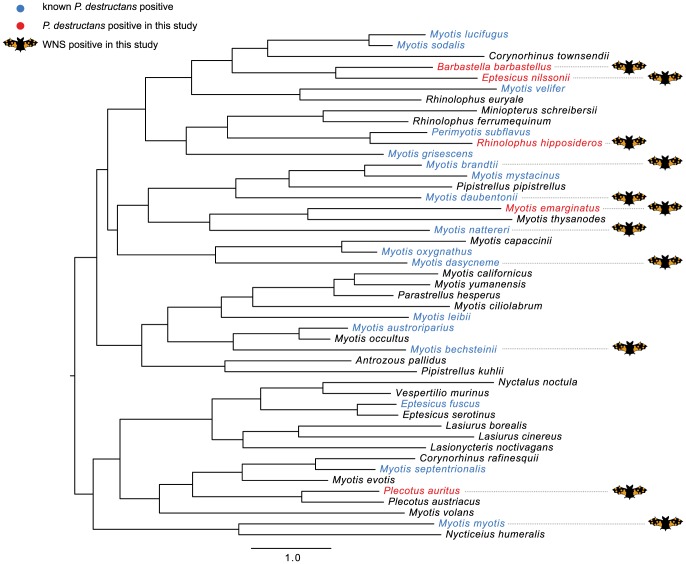
Neighbour-joining tree based on ecological and behavioural traits of bats from Europe and North America (rooted at midpoint). See Figure 2 for a description of the colour scheme.

The ecological similarity tree for European and North America bats was constructed using the squared Euclidean distances of the traits dataset (Figure 3). Analysis of *P. destructans*-infected species distribution indicated that infected species were randomly distributed (MPD  =  7.839, NRI  =  0.862, *p*  =  0.201) across the ecological diversity of bats from these two continents. The most ecologically similar species were also infected randomly (MNTD  =  4.356, NTI  =  0.277, *p*  =  0.384; Table 2, Figure 3).

#### Predicting species at risk from *P. destructans* infection

We explored relationships between ecological traits after removing the effects of bat species relatedness using PGLS. The final model, displaying lowest AIC (AIC  =  69.08, *F*-statistic  =  8.98, df  =  7 and 39, *p* < 0.001, adjusted *R* ^2^  =  0.55), differed from two more complex models by ΔAIC < 3 (Table S3). The addition of the variables did not markedly alter results of the analysis as reported below, and therefore we used the model with the lowest AIC. It describes the relationship between *P. destructans* infection in bat species and Temperature preference during hibernation (*β*  =  −0.207, SE  =  0.085); Roost Shelter during hibernation: Hidden (*β*  =  −0.109, SE  =  0.211), Exposed (*β*  =  0.560, SE  =  0.192); Cluster Size during hibernation: Small (*β*  =  −0.386, SE  =  0.130), Medium (*β*  =  −0.309, SE  =  0.194); Distribution range size: Moderate (*β*  =  −0.201, SE  =  0.089); and Feeding habitat: Closed (*β*  =  0.319, SE  =  0.113). Shapiro-Wilks' normality test indicated that model residuals were normally distributed (W  =  0.981, *p*  =  0.63).

**Figure 4:**
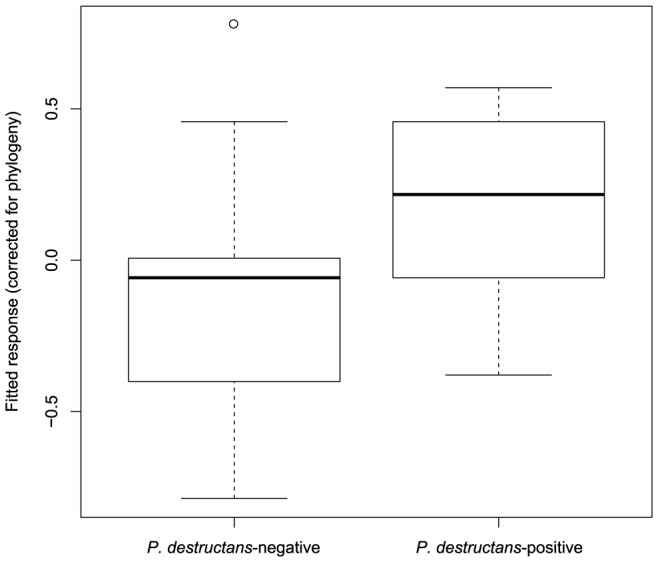
Boxplot of fitted values from the phylogenetic generalised least squares model of *P. destructans* infection. Predictions for infected and non-infected species overlap.

The fitted values of *P. destructans* infection in bats based on the PGLS model showed overlap between the *P. destructans*-positive and -negative bats (Figure 4). Bat species currently recognised as *P. destructans*-negative with highest fitted PGLS values were (in descending order): *Corynorhinus townsendii*, *Lasiurus cinereus*, *Plecotus austriacus*, *Rhinolophus ferrumequinum*, and *Miniopterus schreibersii*.

## Discussion

At the end of the hibernation seasons of 2012 and 2013, we screened 276 bats of unknown infection status and biopsied all bats with WNS-suspected skin lesions. Both the number of bats and the number of taxa (*n*  =  15) examined make this the most extensive and species-rich study of *P. destructans* infection in Europe to date. Earlier European studies have provided data from bats originally sampled for fungal microscopy, culture and genetic analysis when they exhibited obvious fungal growth during hibernation, numbers of species examined ranging from 1 to 12 and numbers of specimens from 1 to 107 [14], [15], [19]–[22].

This study documented five additional bat species as positive for *P. destructans* infection and added ten species from the genera *Myotis*, *Eptesicus*, *Plecotus*, *Barbastella* and *Rhinolophus* to the list of European bat species showing histopathological findings consistent with WNS [23]. Species-specific prevalence of WNS-diagnostic skin lesions ranged from 0 to 100% (4–55% for species with *n* > 20; Table 1). Highest prevalence of WNS lesions was observed in *M. myotis* (after excluding species for which just one specimen was caught, i.e. *M. dasycneme*). Note, however, that with prevalence differing by an order of magnitude, detection of positive specimens may have been biased by small sample size in rarer species and species less frequently visible in hibernacula. With our experimental design (i.e. trapping of winter survivors leaving hibernacula and non-destructive UV fluorescence screening), we were able to confirm that *M. myotis* had highest prevalence of WNS lesions (based on histopathology), with the possible exception of *M. dasycneme*. Our results confirmed WNS skin lesions in 11 bat species, which is in contrast to a previous study that found no invasive growth of *P. destructans* in European bats [38]. The latter study, however, examined a low number of bats and used skin biopsy methods lacking in sensitivity and this may explain the failure to detect lesions diagnostic of WNS.

Bat species with *P. destructans* infection exhibited diverse hibernation behaviours. Among these, *R. hipposideros* was special as it is an exclusively solitary hibernator and hangs free in exposed places with the wing membranes covering the body. It is capable of hibernation at higher temperatures but requires the microclimate stability ensured by using the inner parts of caves [39]. *Rhinolophus hipposideros* is also the most abundant species in winter-monitoring counts, amounting to more than 50% of all bats registered in Czech hibernacula (unpublished data, Czech Bat Conservation Trust). Moreover, as a member of the suborder Pteropodiformes, it is phylogenetically distantly related to all other species infected by *P. destructans* (Figure 2). As documented by the low infection prevalence (3.57%), environmentally mediated indirect and density-dependent transmission probably does not result in higher risk for this rhinolophid species [10]. Similarly, *E. fuscus* and *M. leibii*, both solitary hibernators from North America, were the least impacted species. In comparison, higher declines were observed in large winter colonies of two species that roost solitarily or in small groups, i.e. *P. subflavus* and *M. septentrionalis* [10]. Disease risk, however, was not related to conspecific transmission only. When multiple co-occurring species can host the pathogen, density-dependent transmission can be amplified [40], [41].

We confirm here that bat species previously known to be positive for *P. destructans* may later show as WNS-positive based on histopathology. As the lists of bats positive for *P. destructans* or WNS lesions in North American and European species are nearly equal, conclusions drawn from analysis of infection or disease risk should be similar. Traits describing ecological and behavioural characteristics of bats occurring within the known distribution of *P. destructans* indicate that species belonging to additional genera may also be found positive for the infection in the future. Our screening of Czech underground hibernacula, however, demonstrates that the initial, relatively low, number of bat species positive for *P. destructans* infection is more likely the result of sampling bias than a biological phenomenon. Currently, affected species are ecologically diverse, to the point where predictions for infected and non-infected species overlap. We therefore assume that more species will be revealed as WNS-positive with increased sensitivity of detection methods [42].

Phylogenetic representation of *P. destructans* infection indicates that closely related species are most likely to be infected. In terms of field surveys, therefore, some *Myotis* bats are universally likely to be WNS-positive and most effort should be devoted to these species. Interestingly, *M. alcathoe* was free of WNS-positive skin lesions in the present study (but note the low sample size). *Myotis* species typically form clusters during hibernation and such behaviour promotes frequency-dependent transmission of the infection, independent of population size, and may yet drive the species to extinction [9] unless they change their social behaviour, as documented in *M. lucifugus* and *M. sodalis* [10]. In light of our new data, clustering behaviour is not a descriptor common to infected species. Rather, a suite of characteristics, including cluster size, type of shelter during hibernation, temperature at hibernation, as well as size of the distribution range and feeding in closed habitats, play a role in characterising bats with *P. destructans* infection.

Based on the list of species currently known to be affected by WNS or *P. destructans*, it is clear that the fungus is neither species-, genus- nor family-specific. The multi-host occurrence of the pathogen might make the disease less predictable using ecologically- and phylogenetically-based analysis [43]; however, this is likely to change in the future as additional species are revealed as susceptible. Hibernation in contaminated caves and mines under conditions favourable for fungal growth [5], [44] appears to be the main risk factor [4], [12]. Distribution of *P. destructans* is also correlated with disease in hibernating bats [45]. Importantly, 25 species of insectivorous bats presently hibernate in the United States and Canada, all of which represent possible hosts of the fungal pathogen should the disease spread to their geographic range [46]. This scenario is predicted to happen in most counties with caves in the contiguous United States by the winter of 2105-2106 [47].

The reason bats in North America have been so hard-hit, with millions dying, while bats in Europe apparently cope better with the infection, has not yet been explained. Likewise, the pathogenesis of WNS still remains unclear [12]. Behavioural aberrations, physiological disruption and immunosuppression during hibernation are, however, considered key pathomechanisms [4], [48], [49]. On the other hand, restoration of immune responses in WNS-positive bats early in post-hibernation may result in immune-mediated destruction of infected tissues and death [50].

The fact that our samples were mostly collected from bats emerging from hibernacula at the end of the hibernation season indicates that European bat species can survive *P. destructans* infection and highlights the need for a comparison of European and North American bat population responses to this fungal disease. As all European bat species are strictly protected and any thorough pathological study of *P. destructans* infection would be controversial [18], [22], implementation of non-lethal sampling methods is necessary, such as the wing membrane biopsy used in this study [42]. While a detailed comparison of histopathological findings in European and North America bats represents a valid approach to the better understanding of WNS mortality [17], [48], it was outside the objectives of this ecological study. We are however, planning a comprehensive study to investigate the extent of WNS wing lesions in hibernating bats from the two continents.

## Conclusions

This hypothesis-driven study explored clustering of *P. destructans* infection in relation to chiropteran ecological and behavioural trait variation and phylogeny and supported this with field data. Extension of the surveillance to a broader number of species to test the study's hypotheses identified multiple European species positive for WNS. The increased number of positive bat species resulted in random dispersion of *P. destructans* infection across trait trees and weakened the pattern of phylogenetic clustering of *P. destructans* infection. Distantly related bat species, characterised by diverse life histories, were infected and all hibernating bats may, therefore, be at risk from *P. destructans* infection. Ecological and evolutionary constraints on hibernating bats do not pose a barrier to this generalist fungal pathogen, with WNS occurring in both suborders of bats. Our findings indicate that a wider focus is needed in studying the ecology and epidemiology of this fungal disease of major conservation concern.

## Supporting Information

Table S1 **Ecological and behavioural traits of European and North American bat species.** The dataset includes the most common behavioural or trait value for each bat species.
(XLSX)

Table S2 **Accession numbers for phylogeny reconstruction.** A total of 13 available mitochondrial and nuclear genes were used for phylogeny reconstruction of bat species from Europe and North America.
(XLSX)

Table S3 **Phylogenetic generalized least squares model selection.** The model predicting *P. destructans* infection based on ecological and behavioural characteristics of bats was selected with the step-down procedure, where the full model is given on the first line and removed variables are listed subsequently.
(PDF)
