# Supplementary material for: White-Nose Syndrome Fungus: A Generalist Pathogen of Hibernating Bats
Source: PLoS One. 2014 May 12;9(5):e97224. doi: 10.1371/journal.pone.0097224 (PMC4018256; doi:10.1371/journal.pone.0097224)
Supplement: Table S3 — Phylogenetic generalized least squares model selection. The model predicting P. destructans infection based on ecological and behavioural characteristics of bats was selected with the step-down procedure, where the full model is given on the first line and removed variables are listed subsequently. (PDF) [file pone.0097224.s003.pdf]

Table S3. Phylogenetic generalized least squares model selection.
The model predicting *P. destructans* infection based on ecological and behavioural characteristics of bats was selected with the step-down procedure, where the full model is given on the first line and removed variables are listed subsequently.

**Model**	**ln*L***	**AIC**	**AICc**
Pd ~ CAVE + REGION + CLUSTER + TEMPERATURE + SHELTERhidden + SHELTERexposed + CSIZEsmall + CSIZEmedium + RANGEverylarge + RANGElarge + RANGEmoderate + FOODcolleoptera + FOODdiptera + FOODgeneralist + FOODlepidoptera + HABITATclosed + HABITATopen + BODYsmall + BODYmedium	-21.1948	82.39	114.70
~.-BODYmedium	-21.2146	80.43	108.58
~.-BODYsmall	-21.2856	78.57	103.00
~.-FOODlepidoptera	-21.4081	76.82	97.92
~.-FOODgeneralist	-21.9462	75.89	94.03
~.-HABITATopen	-22.1580	74.32	89.80
~.-FOODdiptera	-22.1585	72.32	85.44
~.-FOODcolleoptera	-25.2637	76.53	87.56
~.-CLUSTER	-25.3162	74.63	83.81
~.-REGION	-25.5279	73.06	80.60
~.-RANGElarge	-25.6031	71.21	77.32
~.-CAVE	-26.0697	70.14	75.00
~.-RANGEverylarge	-26.5378	69.08	72.87
~.-TEMPERATURE	-29.8421	73.68	76.56
~.-RANGEmoderate	-31.1150	74.23	76.33
